# A Rare Case of an Artery Passing through the Median Perforating Canal of the Mandible

**DOI:** 10.1155/2016/8183565

**Published:** 2016-04-26

**Authors:** Joe Iwanaga, Koichi Watanabe, Tsuyoshi Saga, Yoko Tabira, Koh-ichi Yamaki

**Affiliations:** Department of Anatomy, Kurume University School of Medicine, Kurume, Fukuoka 830-0011, Japan

## Abstract

Along with the popularization of dental implant surgery, there has been considerable research on the lingual foramen using cone-beam computed tomography. Anatomical research has also revealed that the arteries entering the lingual foramina are branches of the submental and sublingual arteries. There have been no reports, however, of the submental or sublingual artery entering the mandible from the lingual foramen, perforating it, and then distributing to the inferior labial region. A 69-year-old man who donated his body to our department in 2015 was dissected. The mandible with overlying soft tissue of the mental region was resected and examined with microcomputed tomography, which showed that the canal perforated from the lingual foramen to the midline of the labial cortical plate. The canal was thus named the median perforating canal. To the best of our knowledge, there have been no other reports of a perforating artery of the mandible, so this case is thought to be rare. Hence, the existence of perforating arteries, such as in the present case, should be taken into consideration in preoperative diagnoses such as for dental implant surgery. Thus, the fusion of anatomical and radiological study is useful and necessary to understand surgical anatomy.

## 1. Introduction

The development of cone-beam computed tomography (CBCT) has contributed to finding many kinds of accessory foramina of the mandibular bone. The lingual foramen is well known as it enters branches of the submental or sublingual artery in the lingual cortical plate of the mandible [1]. Thus, a preoperative diagnosis of the anatomy for implant surgery in the incisive region is important to prevent accidental hemorrhage [2]. The morphology of the accessory foramina and canals could be clarified on CBCT images. The replacement site for the implant fixture should sometimes be modified to avoid injuring neurovascular bundles passing through those accessory foramina. The course of the artery outside the mandible, however, cannot be visualized by CBCT, and thoughtless handling in the soft tissue near the foramina has a risk of damaging arteries. Both the course of the artery before entering the mandible from the lingual foramen and the branches of the submental artery around the mental region have been studied in detail by gross anatomical dissection [1, 3]. Report of an artery perforating the mandible from the lingual to the labial cortical plate should be taken into consideration during surgical procedures.

Here we report a case of a perforating canal of the mandible, with an artery passing through the canal and distributing to the inferior labial region, which we confirmed anatomically and radiologically. This study was performed in keeping with the requirements of the Declaration of Helsinki (64th World Medical Assembly, Fortaleza, Brazil, October 2013).

## 2. Case Presentation

**Figure 1:**
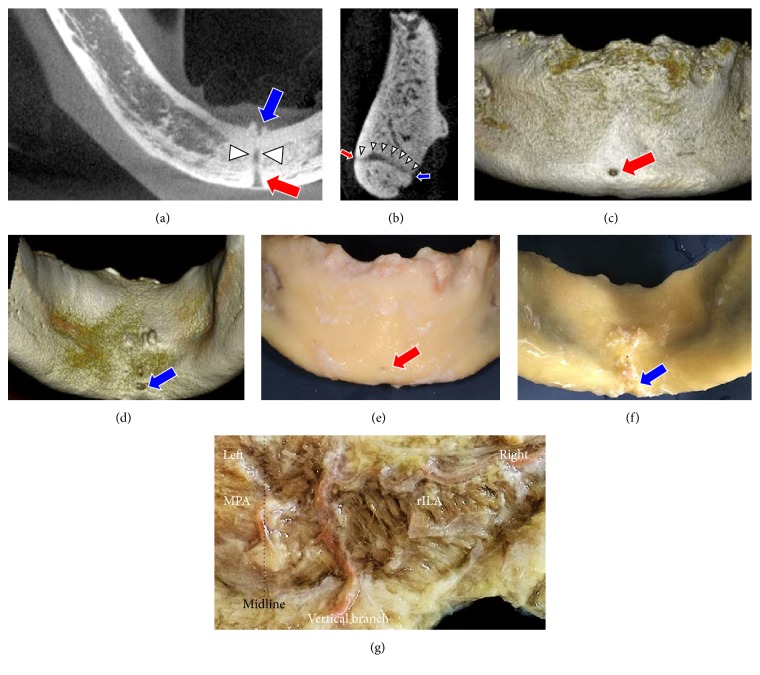
Axial (a) and sagittal (b) images of the mandible show two foramina located on the anterior (red arrow) and posterior (blue arrow) surfaces of the midline. A canal (white arrowheads), which has the continuity of those two foramina, was demonstrated. Three-dimensional computed tomography images of the mandible show anterior (c) and posterior (d) views of the mandible. After removing the overlying soft tissue, the mandible is seen in anterior (e) and posterior (f) views. The medial view of the dissected soft tissue after periosteal detachment shows the right ILA running horizontally and joining the vertical branch, which passes by the inferior border of the mandible (g). The median perforating artery (MPA) has no anastomosis with other branches. Red arrow, median labial foramen (MLaF); blue arrow, median lingual foramen (MLiF); rILA, right inferior labial artery.

A 69-year-old Japanese man who died of cervical esophageal cancer donated his body to our medical school in 2015. An edentulous mandibular bone with the lateral area of overlying soft tissue was resected from his body and examined using microcomputed tomography (*μ*CT) and anatomical dissection. The axial and sagittal images showed two small foramina and a canal connecting the two foramina near the inferior border of the mandible in the midline (Figures 1(a) and 1(b)). Three-dimensional reconstructed images showed that, in the frontal and posterior views, the labial and lingual accessory foramina were located near the inferior border of the mandible in the midline (Figures 1(c) and 1(d)).

**Figure 2:**
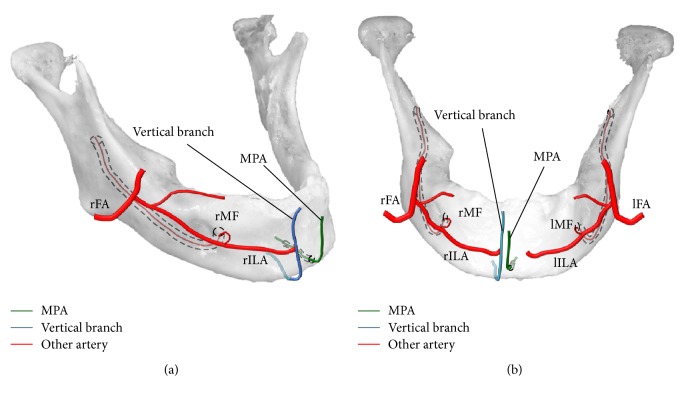
MPA (green line), vertical branch (blue line), and other arteries (red lines) are shown in lateral (a) and frontal (b) views. Both facial arteries branch off the inferior labial arteries (ILAs). Both ILAs anastomose with the mental arteries arising from the mental foramina. The right ILA anastomoses with the vertical branch coming from the inferior border of the mandible without perforating the mandible. The median perforating artery (MPA) comes from the posterior of the mandible and perforates the mandible. It then goes up to the inferior labial region. lFA, left facial artery; lILA, left inferior labial artery; lMF, left mental foramen; rFA, right facial artery; rILA, right inferior labial artery; rMF, right mental foramen.

The labial and lingual foramina were named the median labial foramen (MLaF) and the median lingual foramen (MLiF). The canal connecting the MLaF and MLiF was named the median perforating canal (MPC). The MPC was 9.2 mm long. The MLaF (1.1 × 1.2 mm) was located in the midline and 5.8 mm superior to the inferior border of the mandible. The MLiF (0.9 × 0.9 mm) was located in the midline and 3.6 mm superior to the inferior border of the mandible and inferior to the mental spine. Gross anatomical dissection was carried out to visualize the structure passing through the MPC. The overlying soft tissue was separated from the mandible (Figures 1(e) and 1(f)), and a structure was seen arising from the MLaF. Dissection revealed that only the artery passed through the MPC. It was named the median perforating artery (MPA). The MPA went up to and fed the medial inferior labial region. The vertical branch, which passed by the inferior border of the mandible, also went into the inferior labial region 3.0 mm to the right of the MPA and then anastomosed to the right inferior labial artery (rILA). It was difficult to dissect these thin arteries, so colored derivatives of contrast medium (Microfil red; Flow Tech, Hartford, CT, USA) were injected from both sides of the facial arteries (Figure 1(g)). The contrast medium revealed that the MPA had no anastomosis and directly fed the inferior labial region. The vertical branch went up and joined the rILA and fed the inferior labial region (Figures 2(a) and 2(b)).

In the present case, the mandible was resected from the head by cutting off the origin of the oral floor and tongue, which was on the medial side of the mandible. Hence, the origins of the MPA and the vertical branches were unknown, although they are thought to arise from the submental or sublingual artery.

## 3. Discussion

Bone morphology is easy to understand using CT images. Popularization of implant surgery has caused many more dentists to use CT, appreciating its high resolution that allowed us to visualize the accessory foramina. Cases of severe bleeding caused by injury to the lingual foramen have been reported. Wang et al. investigated the lingual and lateral lingual foramina to learn their epidemiology and morphology [4]. They researched the lingual foramen using CBCT, so the components of the neurovascular bundles were not seen. CBCT showed that 13.2% of the mandible had the lingual foramen in the midline which was >1 mm in diameter.

It is well known that the major nerve in the mental region is the mental nerve, which arises from the mental foramen. Iwanaga et al. [5] noted that the large accessory mental foramen, which was distant from the mental foramen, might contain an artery. Nakajima et al. [1] classified the artery that entered the median lingual foramen based on the patterns of the anastomosis of the sublingual and submental arteries. In the present case, the MPA entering the MLiF was thought to be a branch of the submental or sublingual artery. The artery perforated the mandibular bone and arose from the MLaF and then fed the inferior labial region without any anastomosis. Masui et al. also classified the course of the sublingual artery [6]. The artery passed by the inferior border of the mandible and in this case was thought to be the vertical labiomental artery proposed by Kawai [3]. Smartt Jr. et al. [7] stated that the blood supply to most of the postnatal developing mandible was via the inferior alveolar artery and the periosteal plexus provided by the terminal branches of the lingual and facial arteries. An anastomosis between the inferior* alveolar* artery branches and the submental artery has also been reported [8]. Many arteries fed the mandible during the development process, and the MPA was considered to be a remnant of the feeding artery of the mandible and its surrounding tissue. Embryologically, when both mandibles fused with the midline, it is possible that the bone formation around the MPA created the MPC.

The MPA matters clinically during dental implant surgery, and periosteal detachment of the mandible in the submandibular region must be corrected. CBCT makes it easy to diagnose those accessory foramina preoperatively. In plastic surgery, during a facelift or submental procedures, the submental artery is known to bleed. Hwang et al. explained that branches of the submental artery pass by the inferior border of the mandible, causing the submental artery to bleed [9]. When the submental region is touched during those procedures, variations in the vascular system and bone morphology should be taken into consideration.

This was a rare case of a perforating artery passing through the lingual foramen of the mandible. It is important to remember that findings from anatomical and radiological studies are useful clinically.
